# Emerging Contact-Killing Antibacterial Strategies for Developing Anti-Biofilm Dental Polymeric Restorative Materials

**DOI:** 10.3390/bioengineering7030083

**Published:** 2020-07-30

**Authors:** Heba Mitwalli, Rashed Alsahafi, Abdulrahman A. Balhaddad, Michael D. Weir, Hockin H. K. Xu, Mary Anne S. Melo

**Affiliations:** 1 Program in Biomedical Sciences, School of Dentistry, University of Maryland, Baltimore, MD 21201, USA; hmitwalli@umaryland.edu (H.M.); rashed.alsahafi@umaryland.edu (R.A.); aabalhaddad@umaryland.edu (A.A.B.); mweir@umaryland.edu (M.D.W.); 2 Department of Restorative Dental Sciences, College of Dentistry, King Saud University, Riyadh 11451, Saudi Arabia; 3 Department of Restorative Dental Sciences, College of Dentistry, Umm Al-Qura University, Makkah 24381, Saudi Arabia; 4 Department of Restorative Dental Sciences, College of Dentistry, Imam Abdulrahman bin Faisal University, Dammam 34212, Saudi Arabia; 5 Department of Advanced Oral Sciences and Therapeutics, School of Dentistry, University of Maryland, Baltimore, MD 21201, USA; 6 Center for Stem Cell Biology; Regenerative Medicine, School of Medicine, University of Maryland, Baltimore, MD 21201, USA; 7 Marlene and Stewart Greenebaum Cancer Center, School of Medicine, University of Maryland, Baltimore, MD 21201, USA; 8 Division of Operative Dentistry, Department of General Dentistry, School of Dentistry, University of Maryland, Baltimore, MD 21201, USA

**Keywords:** antibacterial agents, polymers, dental caries, biofilms, composite resins

## Abstract

Polymeric materials are the first choice for restoring tooth cavities, bonding tooth-colored fillings, sealing root canal systems, and many other dental restorative applications. However, polymeric materials are highly susceptible to bacterial attachment and colonization, leading to dental diseases. Many approaches have been investigated to minimize the formation of biofilms over polymeric restorative materials and at the tooth/material interfaces. Among them, contact-killing compounds have shown promising results to inhibit dental biofilms. Contact-killing compounds can be immobilized within the polymer structure, delivering a long-lasting effect with no leaching or release, thus providing advantages compared to release-based materials. This review discusses cutting-edge research on the development of contact-killing compounds in dental restorative materials to target oral pathogens. Contact-killing compounds in resin composite restorations, dental adhesives, root canal sealers, denture-based materials, and crown cements have all demonstrated promising antibacterial properties. Contact-killing restorative materials have been found to effectively inhibit the growth and activities of several oral pathogens related to dental caries, periodontal diseases, endodontic, and fungal infections. Further laboratory optimization and clinical trials using translational models are needed to confirm the clinical applicability of this new generation of contact-killing dental restorative materials.

## 1. Introduction

Biofilms are a complex of microorganism aggregates associated with bacterial cells adhering to each other in an enclosed polymeric extracellular matrix [1]. Biofilm formation on synthetic surfaces represents a significant health problem in industrial and healthcare applications [2]. In medical applications, bacterial attachments on devices and implants have adverse effects on their functionality, restrict the lifespan of such appliances causing bacterial infections, substantially complicating situations in clinics, and in some cases, lead to death [3].

Dysbiotic biofilms are the driver for the majority of oral infections, where dental caries (tooth decay) are considered the most prevalent. Dental caries is increasing worldwide in children and adults [4]. Around 90% of adults were reported to have experienced dental caries, and 26% to have untreated dental caries [5]. Additionally, older adults face medical and dental challenges, which may amplify the destruction produced by dental caries [6]. In older adults with systematic diseases, the utilization of some medications may induce hyposalivation, which may increase the chances of cariogenic species that cause dental caries [7].

The effects of oral infections triggered by biofilms involving existing restorations include restoration replacement and further loss of tooth structure; periimplantitis leading to implant failure and thereby the costly replacement of implant-supported prosthesis that is associated with bone loss; and orthodontic appliances. Similarly, following root canal therapy, biofilms may persist in the root canal system and may cause re-infections. Therefore, the alteration of biofilm development has become a global health priority [7,8].

Unlike planktonic microorganisms, microorganisms found in dental plaque biofilms have shown tolerance to antibacterial strategies [8]. The oral cavity, with its warm, moist, nutritive atmosphere, provides an ideal environment for microorganisms to grow and proliferate. The complicated interaction between microorganisms, diet, and host leads to colonization of bacteria and the development of pathological biofilms. Pathogenic biofilms attaching on the tooth surface or restorative materials via specific binding proteins are virulence factors in the formation of dental caries [4]. The attached biofilms may increase in volume with a more diverse bacterial community as a result of the continuous neglect of oral hygiene and the prevalence of a sugar-containing diet. Caries-related pathogens within the biofilms, mainly *Streptococcus mutans* and *lactobacillus*, upregulate specific virulence factors to consume the fermentable carbohydrate over the tooth and produce lactic acid. The produced lactic acid subsequently can lead to the demineralization of the tooth structure minerals [9]. 

**Figure 1:**
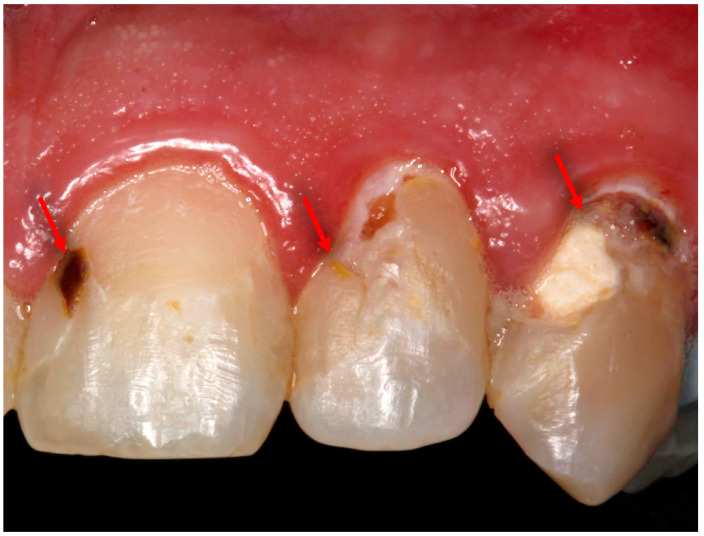
Clinical aspect of several secondary carious lesions around resin composite restorations in the anterior teeth. The arrows in the photo showing the location of the lesions at the tooth/restoration interface presented by yellow and brownish to black discoloration.

Dental composite restorations are the first line of minimally invasive options for the treatment of dental caries in tooth structure. With the increased use of resin-based dental materials, the occurrence of infections related to biofilm is also on the rise. Secondary infections and recurrent caries remain a major problem in the field of restorative dentistry. In previous reports, the prevalence of secondary caries associated with polymeric restorative materials has reached 60%, and it has been recognized as the most common reason for resin composite restorations failure and replacement [10]. The complex structure of the oral cavity with the diversity of microorganisms, the ability of saliva to clear topical antibacterial agents, and increased drug tolerance make topical applications temporary and will render polymeric materials less effective in the long term. Figure 1 illustrates the onset of secondary caries around polymeric-based (resin composite) restorations. The accumulation of plaque at the tooth/restoration interface allows the microorganisms to form biofilms and invade the interface causing another carious lesion.

The recurrent of carious lesions around restorations is under the influence of numerous factors such as the growth of dysbiotic biofilms, difficult access for cleaning between teeth, and surface characteristics of the dental restorative material [11]. A trigger factor for restoration failure is the production of acids from plaque biofilms around the restoration margins [11]. Restoration replacement due failure accounts for 50–70% of all routine restorative procedures. Resin composite restorations have gained dental market dominance due to esthetics and bonding approach, not required extensive retentive tooth preparations [12]. However, the literature point out that some inerent characteristics of resin composite composition and surface properties that promote biofilm growth can be challenge [11].

One strategy to provide a durable, long-lasting restoration is the incorporation of antimicrobial agents to control and/or eliminate these infections. Similarly, any strategy that could disrupt the formation of biofilms would be considered clinically valuable as a route to control infections related to biofilm accumulation. To address these issues, extensive efforts have been focused on developing surfaces with antibacterial properties that can diminish the degree of bacterial attachment initially, thus preventing the formation of biofilms [10,11,13].

**Figure 2:**
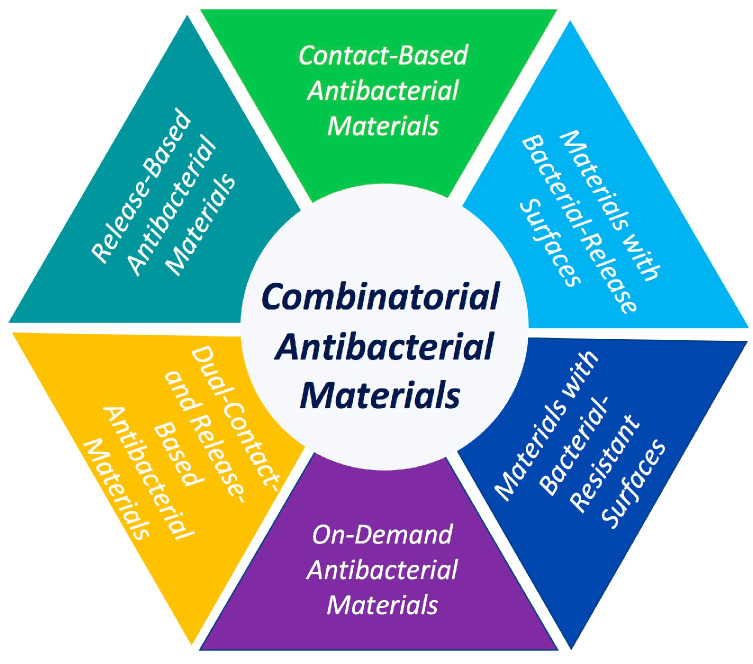
Antibacterial killing strategies imparted in biomedical and dental devices to induce bioactivity against pathogenic biofilms.

To control infections related to biofilm attachment on restorative materials, antimicrobial agents were introduced into dental materials to inhibit microbial adhesion and biofilm formation. Different classes of agents in the development of antibacterial materials are illustrated in Figure 2 [14]. This review provides a critical assessment of newly published contact-killing antibacterial agents’ strategies to control biofilm-related dental infections in dental restorations. 

## 2. Ideal Antibacterial Agents Intended for Dental Restorations

**Figure 3:**
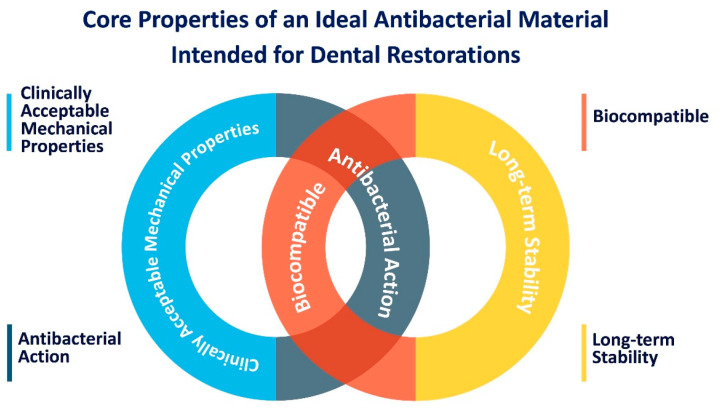
Core properties of an Ideal antibacterial material intended for dental restorations [10,13].

**Figure 4:**
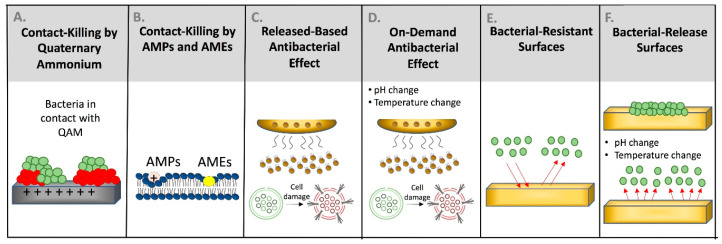
Antibacterial killing strategies can be achieved via different approaches. The contact-killing mechanism can be provided by quaternary ammonium compounds posing highly positive charged surfaces to disrupt accumulated microorganisms (**A**). Antimicrobial peptides (AMPs) and antimicrobial enzymes (AMEs) can conduct contact-killing by invading the cellular membrane and targeting the main cellular components (**B**). Antimicrobial peptides can also conduct antibacterial action via its positively-charged surface. The antibacterial action via ion release can be provided by release-based (**C**) and on-demand (**D**) antibacterial materials. Materials interfering with bacterial adhesion can be designed using bacterial-resistance and bacterial-release surfaces (**E**,**F**).

Antibacterial agents are chemicals that interfere with bacterial reproduction and development, thus reducing the harmful effects of bacteria, as illustrated in Figure 3. Many criteria should be considered when developing resin-based materials with antibacterial properties. An ideal antibacterial agent included in dental restorations would have no undesirable effects on the mechanical properties while maintaining long-term antibacterial benefits without resulting in toxic effects [15]. A strategy to maximize the long-term antibacterial benefits would be to not only have the antibacterial agent limited to the surface but preferably present throughout the entire volume of the material. By doing so, the material may retain the same antibacterial functionality even after wear that may remove the surface of the restoration. Microbial biofilms are a burden for the health care industry that has caused more than 60% of the overall infections acquired through colonization of different therapeutic devices ranging from catheters to dental materials [16]. Therefore, extensive efforts have been focused on developing an antibacterial agent with broad-spectrum activity against cariogenic bacteria and low toxic effects while maintaining clinically acceptable mechanical and antibacterial properties [13]. Many strategies have been developed to impart antibacterial properties in medical devices (Figure 4), which are discussed in the next section.

## 3. Types of Available Antibacterial Strategies

### 3.1. Contact-Based Antibacterial Materials

Non-leaching contact-based antibacterial materials can kill microorganisms and reduce biofilm build-up through antibacterial agents incorporated on the surface by either physisorption or covalent conjugation [2]. Different antibacterial agents are used for this purpose. Quaternary ammonium compounds (QACs), and polycations are examples of synthetic agents while antimicrobial peptides (AMPs), and antimicrobial enzymes (AMEs) are examples of natural antibacterial agents [2,8].

Quaternary ammonium compounds have been developed and incorporated into dental materials. As shown in Figure 4A, the positively charged quaternary amine N+ of QACs acts to change the essential ionic balance of the bacterial cell (i.e., Na+, K+, Mg^2+^, and Mg^2+^, Ca^2+^) and disrupt the membrane by direct binding and interacting with the negatively charged bacterial cell membrane [17]. For quaternary ammonium monomers that are short-chained, the antibacterial activity relies only on an ammonium group that is positively charged. On the other hand, quaternary ammonium compounds with long-chain have double-killing properties: (1) the positively charged quaternary amine N+; and (2) the increased hydrophobicity as a result of the increased alkyl chain length, thus improving its ability to penetrate the hydrophobic bacterial cell membrane [18]. 

Antimicrobial peptides (AMPs) are endogenous biomolecules with broad antimicrobial activity and the ability to kill viruses, fungi, and both Gram-negative and Gram-positive bacteria. These proteins have antibacterial behavior and limited immunogenicity. Plants and insects initially use AMPs as an antibiotic to protect against likely pathogenic microorganisms; however, microorganisms also yield AMPs to protect their environmental position [5]. Approximately forty-five AMPs have been recognized inside the oral cavity, produced by the innate immunity via epithelial cells, gingival crevicular fluid, saliva, and neutrophils [19]. Antimicrobial peptides may be used to avoid inducing bacterial resistance, as is commonly observed in the use of antibiotics [20]. Antimicrobial peptides can be derived naturally or synthetically, depending on many factors related to the nature of such peptide and the intended application. 

Antimicrobial peptides attack Gram-positive microorganisms, bypassing the porous bacterial wall to target the intracellular components (Figure 4B) [21]. The amino acid residues of the AMPs are hydrophobic and positively-charged and may induce specific interactions with the negatively-charged bacterial membrane by competing with calcium and magnesium ions linked to the polysaccharide of the microorganisms (Figure 4B) [22]. The positively-charged AMPs may also be attracted via anionic phospholipids and phosphate groups found over the polysaccharide. Finally, AMPs can change the electrochemical gradient and compromise the bacterial membrane by changing the cellular morphology and damaging the DNA [22].

The use of AMPs in medicine has expanded due to their high efficiency against many microorganisms, including drug-resistant species [23]. The immobilization of AMPs on the surface of medical devices to induce contact-killing has been reported. Antimicrobial peptide coatings on titanium implants and polyurethane catheters have been shown to prevent the adhesion of several pathogens [24]. AMPs are also used to treat skin infections and improve wound healing [25].

In dentistry, AMPs have shown promising results to inhibit the adhesion and growth of several oral pathogens. β-defensin-3 peptide fragment (D1-23) functionalized into a crystalline system was found effective against *S. mutans* showing the potential to inhibit the onset of dental caries [26]. Histatin-derived antimicrobial peptide reduced the *S. mutans*, *Streptococcus salivarius*, *Streptococcus sanguis*, and *Fusobacterium nucleatum* growth by 2–3 logs [27]. Dhvar4 antimicrobial peptide demonstrated effectiveness against anaerobic microbes such as *F. nucleatum*, *Veillonella parvula*, and *Prevotella intermedia* by reducing the growth by approximately 3 logs [27]. Antimicrobial peptides were also found effective against other oral pathogens such as *Enterococcus faecalis*, *Actinomyces naeslundii*, and *Streptococcus gordonii* [28]. For dental restorative applications, several AMPs were functionalized into dental adhesives and resin composite to prevent the growth of caries-related pathogens [26].

Some limitations have been reported with the use of AMPs in medical devices. Most of the AMPs found inside the oral cavity are in concentrations lower than the minimum inhibitory concentration (MIC). Using these AMPs in the MIC level could induce cytotoxic effects by damaging the surrounding tissues. Additionally, AMPs are susceptible to degradation by oral microbes, which may limit the long-term activity of such peptide [29]. 

Antimicrobial enzymes (AMEs) are another example of natural antibacterial agents. AMEs are a group of enzymes that have a significant role in the defense mechanisms of living organisms. They are natural substitutes to the synthetic bactericidal agents targeted to directly disrupt the cellular machinery of bacteria and biofilm formation [16]. To successfully remove complex biofilms, the use of multiple enzymes is required. Antimicrobial enzymes can degrade the DNA of the microbes, their polysaccharides, proteins, and inhibit the quorum-sensing process [16]. AMEs can be chemically immobilized by covalent bonding or physically incorporated by adsorption or self-assembly. They provide a wide-range antimicrobial property at their lowest concentrations with minimal microbial resistance. It was shown that the combination of AMEs with AMPs provides a more significant role for inhibition of biofilms.

Dispersin B is one of the most commonly utilized polysaccharide hydrolyzing enzymes [16]. Previous research studied the anti-biofilm effect of using dispersin B enzyme combined with an antimicrobial peptide and a gelling agent on wound healing and found a significant bacterial inhibition effect [30]. 

Antimicrobial enzymes have other potential benefits, such as the possibility of bactericidal activity combined with highly specific plaque disaggregation and disruption. Moreover, a variety of hydrolytic enzymes of different specificity such as lipase, amylase, and protease have been approved for food use and oral administration [31]. However, some concerns have been raised for AMEs, which include higher cost compared to conventional antibacterial agents and increased risk of infections from the cell dispersal of the biofilm [31]. Therefore, it may be needed to combine AMEs with other antibacterial agents to overcome the risk of infections [16]. 

### 3.2. Release-Based Antibacterial Materials

Release-based antibacterial materials exert their action by releasing preloaded or embedded antibacterial compounds slowly to kill bacteria, as shown in Figure 4C. They can kill both planktonic and attached bacteria. The release of preloaded antibacterial compounds is attained through degradation, hydrolysis of covalent bonds, or diffusion into the environment [32]. 

Release-based antibacterial materials minimize the risk of bacterial resistance and decrease possible adverse systemic consequences by delivering antibacterial compounds only where needed. However, they have a short term efficacy since there is a limited concentration of antibacterial agents present [14]. 

There are many forms of release based antibacterial materials that incorporate nitrogen oxide, antibiotics, or silver nanoparticles (NAg) as releasing antibacterial agents [17]. NAg is the most commonly used release based antibacterial agent [32]. NAg has excellent biocompatibility and a low toxicity level to human cells with robust and broad-spectrum antibacterial outcomes [33]. 

The main mechanism through which NAg exerts its antibacterial effect is by Ag ions disrupting the function of bacterial enzymes, causing the bacterial DNA to lose its reproductive capability, leading to cell lysis and death. Furthermore, NAg has a long-distance killing ability and could kill bacteria, not near the material surface [34]. However, one of the major disadvantages of the incorporation of NAg is its effect on color. Other metallic nanoparticles, such as zinc oxide and copper oxide, as well as calcium phosphate compounds, have been incorporated into dental restorative materials to resist the cariogenic effects of caries-related pathogens [10]. The main concern in this approach is related to materials deterioration following the release of the incorporated agents, which may affect the long-term performance of such materials. 

### 3.3. Dual-Contact- and Release-Based Antibacterial Materials

Contact-based antibacterial agents can be used in combination with other antibacterial agents to improve the antibacterial potency. Combining another antibacterial technique with contact-based antibacterial agents can provide long-term antibacterial efficiency by minimizing bacterial proliferation and resistance. A bioactive material with a dual antibacterial activity should contain: (1) a release-based agent can be delivered into the infection site to eradicate persisting microorganisms; and (2) a contact-based agent that can inhibit microbial proliferation on the surface through contact-inhibition. This dual-action can play a significant role in avoiding treatment complications [17]. 

### 3.4. On-Demand Antibacterial Materials

On-demand antibacterial materials are materials that activate their antibacterial capability when there is a change in the local environment or in response to particular stimuli [35]. Figure 4D illustrates the ability of these materials to release the antibacterial agents in response to a particular trigger due to materials change in structure, volume, or splitting of a chemical bond. Different triggering approaches based on thermal, photothermal, mechanical, and magnetics have been employed [14]. 

Silver nitrate was incorporated into thermo-responsive poly(N-isopropyl acrylamide-co-allylamine) nanogels in a recent study. At 28 °C, bacterial proliferation and growth were documented; however, at 37 °C, the composite collapsed and released silver nitrate, which led to a significant reduction in bacterial load [36]. However, on-demand antibacterial materials are not commonly used. The primary limitations facing these types of materials are controlling non-triggered antibacterial activation and achieving a significant long-term release [14]. The temperature and pH level inside the oral cavity may fluctuate, responding to different conditions rather than bacterial activity. For instance, drinking a beverage that has a low pH may activate such materials when the activation is not required. Continuous undesired activation may exhaust the material and reduce its effectiveness. The same concept could be applied with temperature-dependent materials where the activation can occur with drinking hot or cold beverages.

### 3.5. Materials with Bacterial-Resistant Surfaces

Bacterial-resistant surfaces reduce the early phase of biofilm formation by decreasing the amount of initial bacterial attachment (Figure 4E). Bacterial attachment is facilitated by protein adsorption on the materials’ surfaces, which provides anchor sites for bacterial growth [37]. Surface immobilization of agents that can reduce protein adsorption significantly reduces the amount of bacterial growth [38]. In aqueous environments, these material surfaces can form a physical barrier in the form of a hydration layer. In general, there are two main approaches: ethylene glycol (EG)-based surfaces and zwitterion-based surfaces [39,40].

#### 3.5.1. Ethylene Glycol-Based Surfaces 

Poly(ethylene glycol) PEG is a well-recognized bacterial-resistant material that has minimal protein binding, due to its low surface energy [41]. It is believed that two possible mechanisms are responsible for the protein resistance of PEG-based materials: (1) the compression of PEG chains can produce a repulsive elastic force when proteins adsorb and move towards the substrate surface; and (2) the formation of the hydration layer [42,43]. Incorporating EG-based surfaces in biomedical polymers for wound healing exhibited resistance to cell adhesion and protein adsorption [44]. 

PEG-based agents have been successfully incorporated into dental composites [45]. However, more studies are needed for the long-term mechanical performance, durability, and bacterial resistance ability of such materials to be assessed. Moreover, these materials only decrease bacterial attachment to the material surface with no bactericidal effect. Consequently, physical or chemical factors in the oral environment may deteriorate the PEG coating, leading to biofilm accumulation. Additionally, the limitation of EG-based materials is that they are highly sensitive to oxidation in a biomedical environment. Incorporating other antibacterial agents could help to improve the long-term function of this type of material. Further studies are needed to investigate the long-term stability of these materials and assess their clinical applicability [43]. 

#### 3.5.2. Zwitterion-Based Surfaces 

Zwitterions or dipolar ions are electrically neutral ions with positive and negative electrical charges at different locations within a molecule. Zwitterionic polymers are a distinctive group of smart materials with both positive and negative charges included in their structure [46]. Their bacterial-repelling properties are correlated to the formation of hydration layers’ physical and energetic barrier on the material surface that reduces proteins and bacterial attachment. The hydration layer in zwitterionic materials is more tightly bound to materials surfaces through strong electrostatic interactions than EG-based materials. The EG-based materials hydration layer is weakly bound to materials surface by limited hydrogen bonds [40]. Materials with bacteria-resistant surfaces can reduce bacterial attachment with no interaction or bacterial killing outcome. Therefore, any surface defects or deterioration due to long term physiological or chemical interaction may reduce the surface’s ability to repel proteins and prevent bacterial attachments, rendering it vulnerable to contamination.

### 3.6. Materials with Bacterial-Release Surfaces

Bacterial-release surfaces allow the initial attachment of bacteria and, under a specific environment or condition, can release them from the materials’ surface (Figure 4F) [47]. Bacterial surface attachment is highly dependent on the properties of surfaces. Stimuli-responsive surfaces are a promising type of bacterial release surfaces. Physicochemical changes in stimuli-responsive surfaces can change surface properties from a bacterial-attachment state to a bacterial-repellent state, which leads to complete removal and release of the attached bacteria and biofilm [48]. 

Thermo-responsive polymers are an example of bacterial-release surfaces. Poly(N-isopropyl acrylamide) is the most commonly used thermo-responsive polymer. At low temperatures, poly(N-isopropylacrylamide) allows initial bacterial attachment and biofilm growth. When the temperature is increased, surface characteristics change and repel and release not only newly attached bacteria but also completely mature biofilms [49]. Thermo-responsive polymers have rapid shrinkage kinetics due to temperature change; therefore, using it in the oral cavity may be challenging. Oral cavity temperature increases after each meal due to sustained chewing and increased blood flow to the muscles of mastication [50]. Drinking hot or iced fluids also changes the oral cavity temperature significantly. Uncontrolled shrinkage kinetics of thermo-responsive polymers in the oral cavity could lead to undesirable results. Therefore, using thermo-responsive polymers in the oral cavity may be limited. 

pH-responsive polymers can also be used as a bacterial release material. In an acidic environment, the surface of this type of material is positively charged, resulting in the attachment of negatively charged bacteria. These attached bacteria can be easily liberated when surface charge changes from positive to neutral. The main driving force for this type of surface is electrostatic interaction; it will attract bacteria when it has the opposite charge and repels bacteria when it has the same charge [51]. Many promising strategies in using release-based materials have been implemented [49,51,52,53]. However, more studies are needed to evaluate the mechanical performance and to determine the best approach to improve their long-term stability. 

### 3.7. Dual-Function Antibacterial Surfaces 

Based on the combination of antibacterial agents, there are three categories of dual-function antibacterial materials: kill and release, kill and resist, and resist and release [2]. The first two categories are more appropriate for biomedical applications than the last category, due to the dual action of offensive contact killing mechanism and defensive release or surface resistance mechanisms. The last category has non-biomedical applications [54]. 

Kill and release dual-function antibacterial materials are based on the combination of contact-based killing and bacterial-release action. One drawback of contact killing materials is dead bacterial accumulation, which can provide other microorganisms with a suitable environment for proliferation and compromise the bactericidal efficiency of the contact killing mechanism. Therefore, to maintain long-term antibacterial activity, it is desirable to clean and remove dead bacteria from the material surface [55]. Thermoresponsive polymers and zwitterionic polymers are one of the most common polymers used to kill and release antibacterial approaches [55,56].

The second category of dual-functional antibacterial materials is to kill and resist. This category is based on preventing bacterial attachment to enhance the direct contact killing approach. The combined use of direct bacterial killing and resistance of the initial bacterial attachment resulted in a more reliable and effective bacterial reduction than using each type alone. Significant protein or bacterial attachment on the contact-based antibacterial surface have shown to reduce the antibacterial effect [57]. 

## 4. Why Using Contact-Killing (Non-Leaching) Materials is Preferred Compared to Leached/Released Materials in Dentistry?

Substantial efforts have been made to develop restorative materials with antibacterial properties. Releasing antibacterial agents such as silver, fluoride, and chlorhexidine particles have the advantage of a high level of release without developing resistance or exceeding the systemic toxicity. However, they are dispersed in the matrix, and their effect is diminished over time [10,32]. Similarly, the mechanical properties may decrease over time due to voids formation after release. It was reported that the incorporation of chlorhexidine gluconate at 1% decreased the compressive and tensile strengths of the material [58]. To avoid these adverse effects, contact-killing antibacterial agents covalently-bonded with core dental monomers in the dental formulations were introduced. The copolymerized antibacterial agent exerts its effect through contact-killing without leaching out and thereby offers long-lasting antibacterial benefits. Moreover, there is a limited impact on the curing behavior, and mechanical properties and durability could be sustained after water aging [59,60]. 

## 5. Contact-Killing Materials as a Strategy in Resin-Based Restorative Materials

In general, resin-based materials are inert and have no associated bioactivity. Traditional resin-based dental materials consist primarily of methacrylate monomers. Due to the presence of ester moieties in these methacrylates, they are highly susceptible to degradative stresses caused by bacterial invasion and salivary enzymes [61]. Considering that no resin-based material can be polymerized completely to achieve the 100% conversion of monomers to polymers, the degradation stresses lead to a release of unreacted monomers within the material, which may trigger bacterial attachments and biofilm formation [62]. Therefore, many efforts have been employed to design bioactive resin-based materials that can resist bacterial attachments and neutralize acidity in the oral cavity in order to enhance the clinical longevity of such materials.

**Figure 5:**
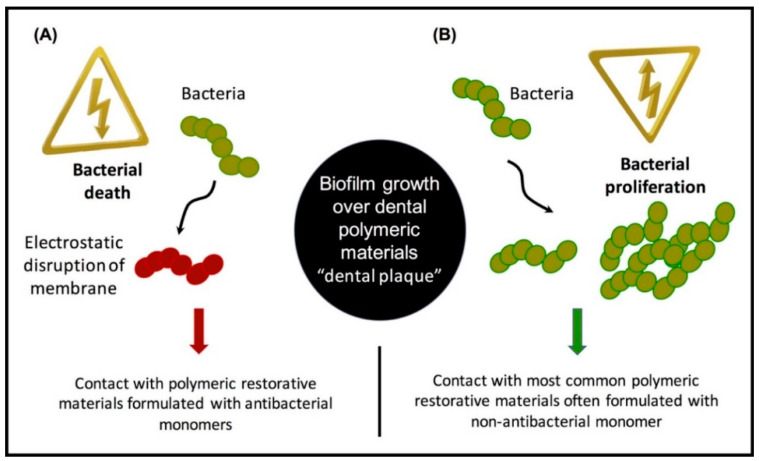
The contact-killing mechanism has been introduced in dental resin composites through the use of antibacterial monomers. Resin composite containing an antibacterial monomer was associated with fewer bacterial colonies (**A**) compared to a conventional resin composite (**B**).

Figure 5 illustrates the differences between antibacterial and non-antibacterial resin composites. In Figure 5A, resin composite containing an antibacterial monomer was effective in reducing the colony-forming units (CFUs) through a contact-killing mechanism. In contrast, a conventional resin composite with no antibacterial compounds showed significant biofilm growth (Figure 5B). Table 1 demonstrates the most commonly used QAMs in resin-based restorative materials, which are discussed comprehensively in the next sections.

### 5.1. Contact-Killing Materials in Dental Resin Composite Restorations

Resin composite restorations are the most popular material used in dental practice because of their excellent esthetics and handling properties [86,87]. Resin composites are criticized for accumulating more plaque compared to other dental restorations such as amalgam and glass ionomer, which can release fluoride, zinc, silver, or copper as bioactive ingredients [88,89]. In fact, resin composite restorations are frequently replaced due to secondary caries at the tooth–restoration interface. However, amalgam restorations are less esthetic and require aggressive cavity preparation compared to resin composite restorations. On the other hand, glass ionomer cements have comparatively inferior mechanical and physical properties [88]. As a result, many efforts have been conducted to incorporate bioactive components in the resin composite system to enhance their antimicrobial performance.

QAMs derive their contact-killing mechanisms by the interaction between the positively-charged surface of QAMs and the negatively-charged bacterial membrane [17]. This concept of immobilized antibacterial monomers was first introduced in 1993 and has attracted much attention since then [90]. 12-methacryloyloxydodecylpyridinium bromide (MDPB) monomer was the first antibacterial monomer to show bactericidal effects against *S. mutans* and other six species of oral *streptococci* [63,91]. 

The effect of QAM alkyl chain lengths on antibacterial response was also investigated. It was found that an increase in the alkyl chain length corresponded to a greater antibacterial response. In the study, dimethylaminohexadecyl methacrylate (DMAHDM) with a 16-unit alkyl chain demonstrated the highest antibacterial properties against caries-related pathogens [18]. Resin composites containing 3% DMAHDM reduced biofilms growth and activities more effectively than the other QAMs without affecting the mechanical properties of the material [67]. DMAHDM incorporated in resin composite restorations significantly inhibited the periodontitis-related pathogens biofilms such as *Porphyromonas gingivalis*, *P. intermedia*, *Prevotella nigrescens*, *Aggregatibacter actinomycetemcomitans*, *F. nucleatum*, and *Enterococcus faecalis* [66].

**Figure 6:**
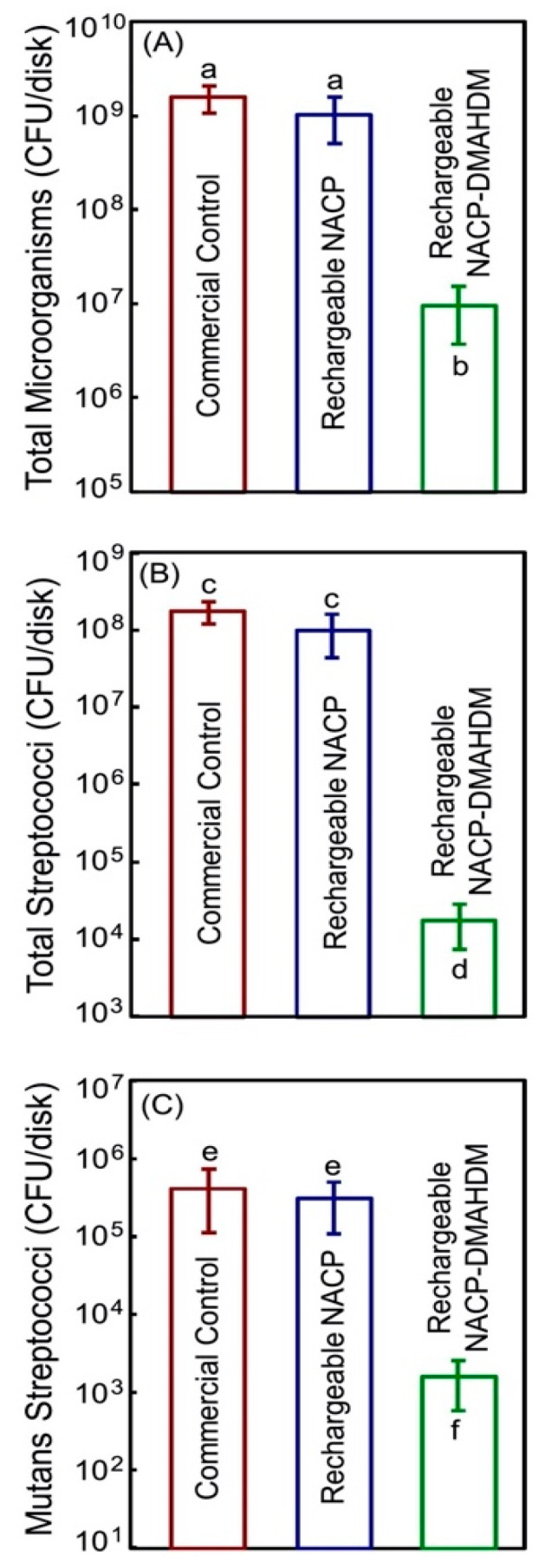
Colony-forming units were grown over the resin composite surfaces using saliva-derived biofilm: (**A**) total microorganisms; (**B**) total *Streptococci*; and (**C**) *Mutans streptococci.* Adapted from Reference [65], with permission from © 2020 Elsevier.

The use of a dual approach to inhibit dental biofilms was attempted in several previous studies. The contact-killing mechanism induced by antibacterial monomers could be supplemented with bioactive fillers to enhance the anti-cariogenic properties of the resin composite formulations. In one study, the composite resin restoration was designed to induce contact-killing action by 3% DMAHDM antibacterial monomer and also to release calcium and phosphate ions to remineralize the tooth structure and neutralize the acidity [65]. The designed formulation also provides rechargeability via specific solutions to release calcium and phosphate ions. Such an approach could be useful to maintain the stability and integrity of the composite restoration [65]. Figure 6 illustrates the ability of this bioactive antibacterial formulation to inhibit the growth of multispecies saliva-derived biofilms by approximately 2 to 4 logs.

**Figure 7:**
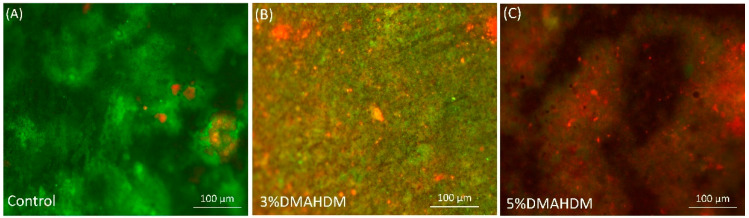
Representative live/dead staining images of a 48 h biofilms grown over different resin composite formulations: (**A**) control; (**B**) 3% DMHADM; and (**C**) 5% DMHADM.

In another study, a resin composite formulation containing 5% DMAHDM was optimized to maintain acceptable mechanical properties with increased antibacterial activity [60]. With and without the incorporation of NACP fillers, 5% DMAHDM was associated with higher antibacterial activity against multispecies biofilms compared to the 3% formulation. In Figure 7B, 3% DMAHDM was found to induce antibacterial killing as fewer viable microorganisms were observed over the surface compared to the control. When the 5% DMAHDM resin composite was used, a significantly more significant antibacterial effect was observed, demonstrated by the presence of more dead microorganisms over the surface compared to the 3% DMAHDM formulation (Figure 7C). Increasing the concentration also was associated with an increased surface charge density, which may explain the increased antibacterial response [60].

**Figure 8:**
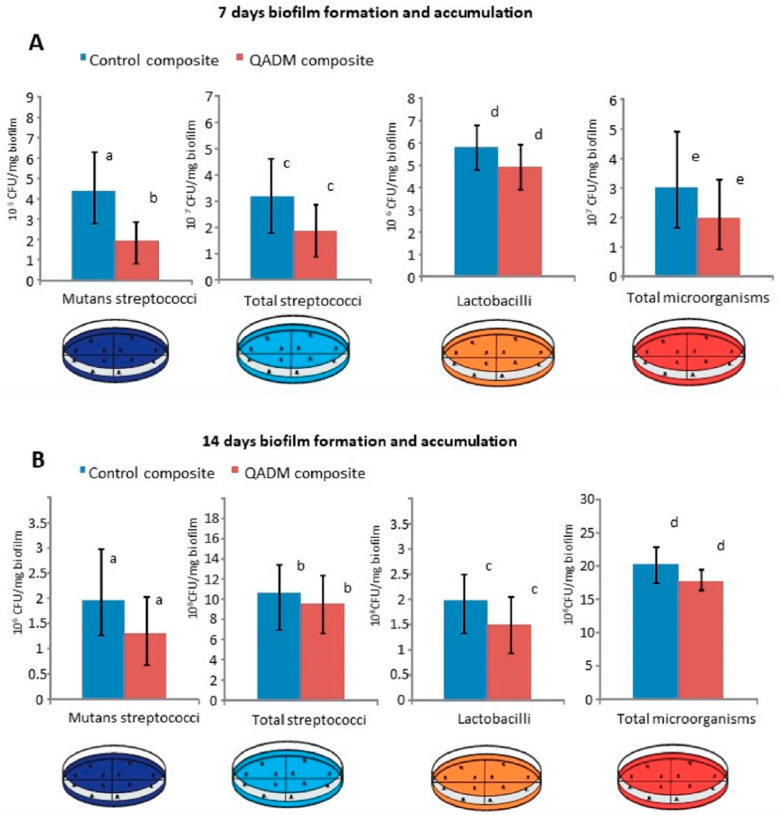
Resin composites containing quaternary ammonium monomers were associated with lower, but not significant, biofilm growth of total microorganisms, total *Streptococci*, *Mutans streptococci,* and total *Lactobacilli* after 7 (**A**) and 14 (**B**) days of biofilm formation in situ. Adapted from Reference [68], with permission Melo et al., 2018.

In a recent study, the antibacterial properties of resin composites containing quaternary ammonium with 12 carbon chain lengh investigated using an in situ model [68]. Patients were asked to wear an intraoral device containing the resin composite specimens, and two weeks later the attached biofilms were collected. Figure 8 demonstrates the quantification of total microorganisms, total *Streptococci*, *Mutans streptococci*, and *Lactobacilli* biofilms 7 and 14 days after wearing the devices. The CFUs of total microorganisms after seven days were reduced significantly in QADM resin composites compared to the control, but no significant inhibition was observed with total *Streptococci*, *Mutans streptococci*, and *Lactobacilli* [68]. After 14 days, even though QADM resin composites had fewer CFUs counts than control, the amount of reduction was not significant. These outcomes suggest that the bacterial biofilm in vivo is more challenging compared to in vitro conditions. Conducting more translational clinical trials using varied formulations and different antibacterial monomers may give a better understanding to overcome the clinical challenges of dental caries.

One of the main limitations of QAMs is the effect of the salivary protein coating. This coating over the restorative material reduces the contact surface area between QAMs and oral microorganisms, which may affect the contact-killing efficiency of these materials. The salivary protein coating can also facilitate the adherence and invasion of cariogenic bacteria [37]. Thus, the incorporation of a protein-repellent agent into the resin composite structure has been suggested to minimize the amount of protein coating on dental materials [92]. 2-methacryloyloxyethyl phosphorylcholine (MPC), a protein-repellent agent, has been used in several studies to reduce protein adsorption and to enhance the contact-killing efficiency of QAMs.

It was observed that 3% MPC in the resin composite system has the strongest antibacterial action without compromising the mechanical properties [93]. Incorporating 1.5% and 3% MPC significantly reduced the protein adsorption over the resin composite surface while providing clinically acceptable mechanical properties [94]. The *Mutans streptococci* biofilms that are involved in caries pathogenesis were reduced by 90% compared to the control resin composites. [94]. 

**Figure 9:**
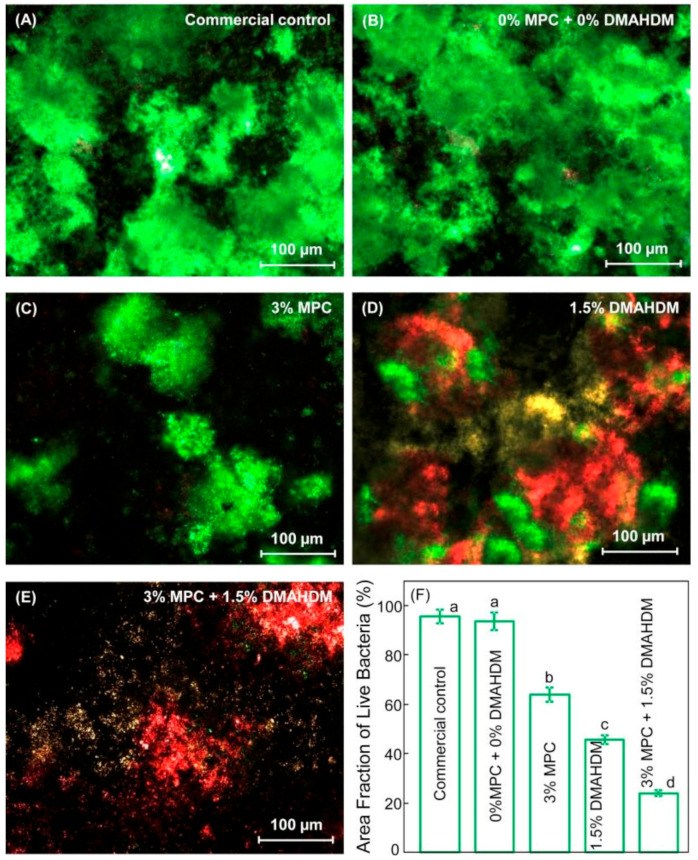
Representative live/dead staining images of biofilms grown over different dental resin composite samples: (**A**) commercial control; (**B**) 0% MPC + 0% DMAHDM; (**C**) 3% MPC; (**D**) 1.5% DMAHDM; (**E**) 3% MPC + 1.5% DMAHDM and (**F**) area fraction of live bacteria. Adapted from Reference [64], with permission from ^© 2020^ Elsevier.

Kill and resist strategy used in a resin composite formulation containing 3% MPC and 1.5% DMAHDM was found to improve the metabolic activities and lactic acid inhibition of the resin composite formulation compared to MPC or DMAHDM alone [64]. The total microorganisms, total *Streptococci*, and *Mutans streptococci* biofilms were reduced by approximately 3 logs, while a 1–2-log reduction was achieved when each agent was used separately. Fewer viable microorganisms were observed over composites containing 3% MPC compared to the control (Figure 9E). 

**Figure 10:**
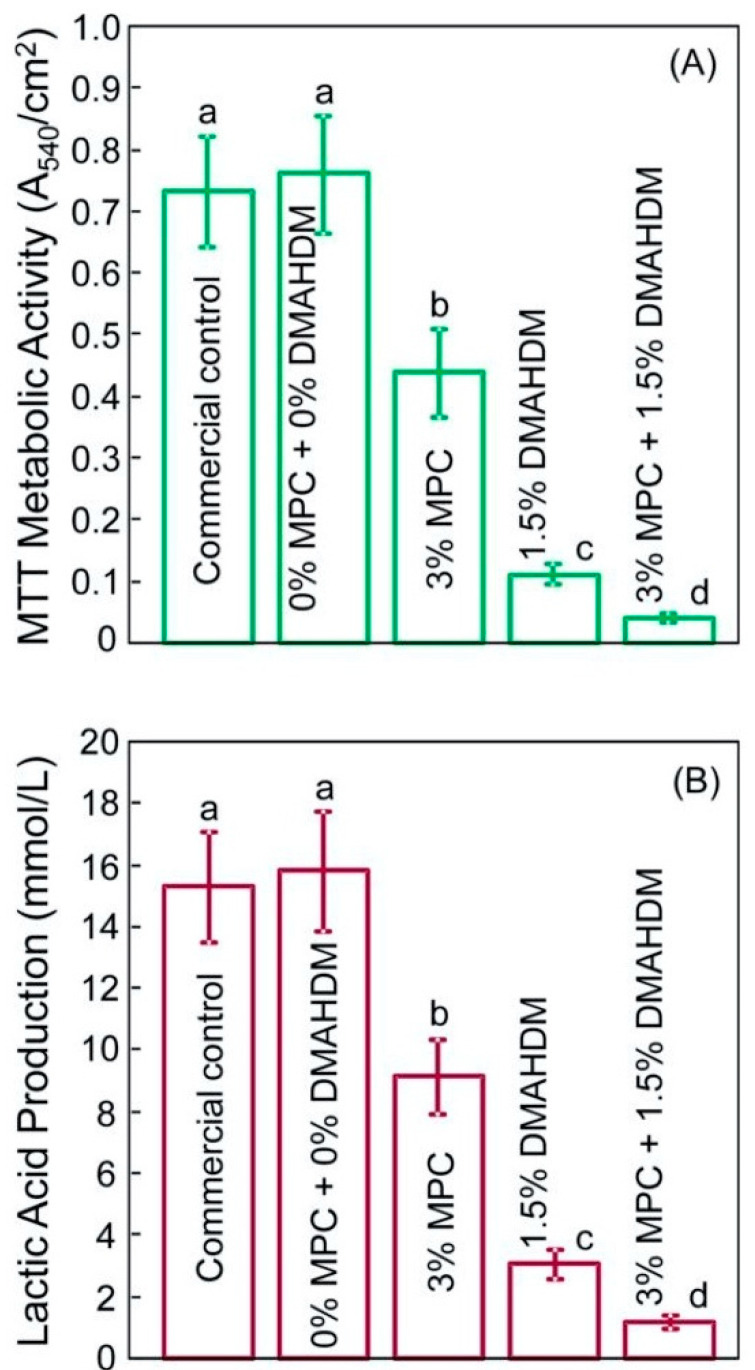
The quantification of metabolic activities (**A**) and lactic acid production (**B**) induced by a multispecies saliva-derived biofilm grown over the resin composite containing an antibacterial monomer. Adapted from Reference [64], with permission from ^© 2020^ Elsevier.

The combination of MPC and DMHADM in dental composites reduced the protein adsorption and also resulted in significant antibacterial action, as shown in the live/dead images in Figure 9D,E [64]. This is also reflected in the significant reduction in lactic acid production represented in Figure 10. This combination provides a dual anti-cariogenic strategy since MPC reduces the attachment of microorganisms by minimizing the protein coating and increases the contact surface area between the DMAHDM and the formed biofilm allowing the contact-killing induction by DMAHDM to be effectively conducted. These results suggest that protein-repellent and contact-killing polymers could be included in the resin composite system to diminish the risk of secondary caries around dental restorations. 

The use of antimicrobial peptides in composite restorations has been reported in one study where Fmoc-pentafluoro-L-phenylalanine-oh (fmoc-f_5_-phe) was functionalized and incorporated into a commercial resin composite at different concentrations: 0.25, 0.5, 1, and 2 w/w % [95]. Fmoc-F_5_-Phe is a self-assembled antibacterial and anti-inflammatory peptide. It has the ability to form hydrogels and induce remineralization. A uniform and even distribution of the peptide nano-assemblies within the resin composite structure was observed via energy-dispersive X-ray spectroscopy (EDX) analysis and optical microscopy. The presence of viable *S. mutans* over Fmoc-resin composites was reduced compared to the commercial control, while the antimicrobial resin composite demonstrated excellent diametral tensile strength and did not show a cytotoxic effect contacting fibroblasts [95].

Future investigations may evaluate the initial and long-term effects of the resin composite containing Fmoc on other types of biofilm as well as mechanical properties such as flexural strength and elastic modulus. More studies are needed to determine the usefulness of AMPs in resin-based restorative materials. Another approach to improve the longevity of resin composite restorations and reduce the risk of secondary caries is by coating the dentin by amphipathic AMPs to create hydrophobic and water-repellent dentin. Such results could be employed to protect the tooth–restoration interface from plaque accumulation and bacterial infiltration [96]. However, it is still unknown if this coating approach is sustainable in protecting the tooth–restoration interface, especially with the degradative effect of host-derived enzymes within the dentin. Further studies are needed to confirm the long-term durability of this approach.

### 5.2. Contact-Killing Materials in Dental Adhesives

Bonding agents (dental adhesives) are used to bond resin composite restorations to the tooth structure [97]. These adhesive resins penetrate etched dentin, infiltrate the exposed collagen, and form a hybrid layer between the tooth structure and resin-based materials allowing the material to adhere firmly without dislodgment or loss of retention [98]. The tooth–resin composite margin at the bonding interface is considered a weak area that is highly susceptible to plaque accumulation and bacterial invasion [98]. Therefore, imparting antibacterial properties in dental adhesives may hinder the growth and activities of caries-related pathogens at the tooth–restoration interface. The use of acidic monomers in self-etching adhesive demonstrated antibacterial effect, but only for the initial 48 h. Another suggested approach has been to incorporate releasing agents such as metallic nanoparticles and chlorohexidine [99]. However, the uncontrolled release and the reduced antibacterial and mechanical properties over time are the main drawbacks of these bioactive releasing agents [15]. 

Therefore, the use of contact-killing materials that can provide a long-lasting effect without leaching has been explored. MDPB was the first antibacterial monomer used in dental adhesives. MDPB provides antibacterial activities against several oral pathogens, including *S. mutans* and *Lactobacilli* species [69,70]. Other monomers such as 2-dimethyl-2-dodecyl-1-methacryloxyethyl ammonium iodine (DDMAI), 2-methacryloyloxyethyl dimethylammonium (IDMA1), and 2,2-bis(methacryloxyloxyethyl dimethylammonium) (IDMA2) were also investigated [71]. The addition of 1–2% of DMAE-CB (methacryloxylethyl cetyl dimethyl ammonium chloride) antibacterial monomer in a dental adhesive significantly reduced the *S. mutans* biofilm, while leaving the micro-tensile bond strength unaffected [100].

The use of MPC has been investigated to explore the effect of reducing protein adsorption over dental adhesives. Incorporating MPC into Scotchbond Multi-Purpose (SBMP, 3M, St. Paul, MN, USA) commercial adhesive reduced the protein adsorption without affecting the bonding strength of the adhesive system [101]. In another study, incorporating 7.5% of MPC and 5% of DMAHDM into SBMP (3M) adhesive system reduced the bacterial load of total microorganisms and *Mutans streptococci* by 3–4 logs and significantly inhibited bacterial metabolic activities and lactic acid production [72]. Another study combined NACP and MPC in a rechargeable dental adhesive system. The calcium and phosphate ion release and recharge were achieved successfully, while protein adsorption was reduced by approximately 10 folds [38]. The total microorganisms, total *streptococci*, and *Mutans streptococci* biofilms were reduced by 1–2 logs compared to control [38].

Using AMPs in dental adhesives have also been studied. In one study, nisin as an antimicrobial peptide was incorporated into a commercial dental adhesive at 1, 3, and 5% (w/v) [102]. Nisin is a cationic, hydrophobic peptide with well-documented antimicrobial properties. A dental adhesive containing nisin reduced the *S. mutans* growth by approximately 2 logs. Fewer colonies were observed using a confocal microscope over the nisin-dental adhesive compared to the parental formulation. All the formulations had a degree of conversion values similar to the control. However, groups with 3% and 5% nisin were associated with lower micro-tensile bond strength compared to the control, while the use of 1% nisin was comparable to the control [102]. 

In a separate study, a dental adhesive containing 3% nisin inhibited a multispecies biofilm and the extracellular polysaccharide production without affecting the degree of conversion and the micro-tensile bond strength [103]. Another study found that the incorporation of GH12, GH12-M1, and GH12-M2 peptides in dental adhesive formulation increased the antibacterial effectiveness against *S. mutans* biofilm [104]. As AMPs are susceptible to degradation, it would be useful for future studies to consider the long-term evaluation of their antimicrobial and mechanical properties. 

### 5.3. Contact-Killing Materials in Endodontics to Prevent Root Canal Reinfection

Several studies have indicated the presence of residual bacteria in the root canal system even after irrigation and root canal instrumentation. The residual biofilms are associated with a risk of developing endodontic re-infection [105,106]. While root canal fillings are supposed to achieve an optimum seal to prevent the growth of the residual bacteria, the complete removal of biofilms is not always possible. Chemomechanical decontamination may not reach areas with limited accessibility, such as isthmus, lateral canals, and apical ramifications, keeping the tooth at a higher risk of re-infection [107]. Therefore, developing new adjunctive strategies to reduce the bacterial load in the root canal system is crucial to enhance the longevity of root canal treatment and minimize the risk of re-infection.

Metallic nanoparticles have been incorporated into root canal sealers to impart antibacterial properties in root canal sealers [107]. The ion release behavior of these materials may not provide a long-lasting effect. As a result, the use of contact-killing materials that can copolymerize with the resin matrix system without leaching has been suggested. The use of quaternary ammonium polyethylenimine (QPEI) nanoparticles was investigated in several studies where QPEI was added in commercially available root canal sealers. Incorporating QPEI into a root canal sealer (Pulp Canal Sealer (PCS) EWT) at 2% weight elicited strong antibacterial response against *Enterococcus faecalis* biofilms [74]. QPEI-PCS reduced the log CFUs of *E. faecalis* by approximately 8 logs compared to the unmodified root canal sealer. However, incorporating QPEI into another root canal sealer (AH Plus) did not demonstrate any additional bacterial reduction compared to the unmodified version of AH Plus [73,74]. 

Incorporating 5% MDPB in root canal sealer reduced the planktonic and biofilm growth of *E. faecalis* by 5 and 2 logs, respectively, compared to control [75]. A recent investigation demonstrated the ability of a root canal sealer containing 5% DMAHDM and 20% NACP to decrease the *E. faecalis* biofilm by 4 logs compared to the unmodified root canal sealer. The bioactive sealer showed sustained release of calcium and phosphate ions, excellent mechanical properties, and achieved functional sealing abilities equivalent to the control [77]. Another sealer containing silver nanoparticles, 5% DMAHDM, and 30% NACP was found to be effective in reducing the *E. faecalis* biofilm on root dentin and recovering the dentin microhardness by approximately 95% compared to sound dentin [76]. 

### 5.4. Contact-Killing Materials in Resin-Based Sealants

Pit and fissure resin-based sealants are commonly used in pediatric dentistry to occlude deep fissures found in posterior teeth to prevent plaque accumulation. Resin-based sealants containing 5% DMAHDM with and without 20% NACP were found effective in reducing the growth of *S. mutans* biofilm while maintaining excellent mechanical properties and recharging capabilities [78,108]. The same formulation also reduced the viability and activities of multispecies biofilms isolated from children with high caries risk and increased the demineralization resistance of the tooth structure [79,80]. 

Another investigation reported the ability of sealant formulation containing 1,3,5-tri acryloyl hexahydro-1,3,5-triazine (TAT) and α-tricalcium phosphate (α-TCP) to reduce the *S. mutans* biofilm by approximately 1.5 logs [81]. The antibacterial sealant formulation demonstrated comparable mechanical properties and higher ultimate tensile strength compared to the control [81]. The use of [2-(methacryloyloxy)ethyl] trimethylammonium chloride (METAC) antibacterial monomer in the sealant formulation was also associated with a 1-log reduction of *S. mutans* biofilms [82], which could be effective in minimizing biofilm formation in teeth with deep pits and fissures.

### 5.5. Contact-Killing Materials in Denture Base Materials

The adhesion of oral biofilm to denture base materials used in full-arch and partial removable dentures may lead to several oral complications such as oral stomatitis, candidiasis, and dental caries in teeth-supported dentures. Most of the denture base materials are made of poly(methyl methacrylate) resin (PMMA), which is highly vulnerable to bacterial attachment and colonization. Thus, the use of contact-killing materials is an essential strategy to eliminate microbial attachment [109]. Incorporating the MDPB monomer into an acrylic denture base reduced the adhesion of *Candida albicans* [109]. Using dimethylaminododecyl methacrylate (DMADDM) in acrylic denture base formulation showed effectiveness against a multispecies biofilm composed of *C. albicans*, *S. mutans*, *S. sanguinis*, and *A. naeslundii*. Incorporating 3.3% of DMADDM reduced biofilm biomass significantly. Furthermore, this antibacterial acrylic denture base material inhibited two virulence genes and prevented the hyphal development of *C. albicans* [83].

### 5.6. Contact-Killing Materials in Orthodontic and Crown Cements

Orthodontic brackets used for dynamic tooth movement act as a plaque stagnation area, facilitating the formation of biofilms and the onset of demineralization at the bracket–tooth interface. These brackets are attached to the tooth surface via orthodontic dental cements. Imparting antibacterial properties into orthodontic cements has demonstrated the potential to minimize the risk of white spot carious lesion formation around orthodontic brackets.

Resin-modified glass ionomer (RMGI) containing 1.5–3% of MPC was effective in reducing the protein adsorption by 3–4 folds, and the biofilm viabilities of *S. mutans* were significantly inhibited [110]. MPC and DMAHDM were combined with silver nanoparticles in a commercially available RMGI cement and found to be effective in reducing the growth and activities of caries-related pathogens. Incorporating MPC and DMAHDM resulted in a 2.5-log reduction of the total microorganisms, total *Streptococci*, and *Mutans streptococci*. Adding silver nanoparticles also resulted in a 3-log reduction [85]. Meanwhile, the use of 1–5% of 2-methacryloxylethyl hexadecyl methyl ammonium bromide (MAE-HB) monomer in orthodontic adhesive resulted in 1–2-log reduction at baseline and 180 days after water aging without compromising the bonding strength. Fewer *S. mutans* colonies were observed over specimens containing MAE-HB compared to the control [100].

Marginal discrepancies at the crown–tooth interface are plaque stagnation areas that facilitate the attachment of oral bacteria and the formation of biofilms, which by then can lead to secondary caries and crown failure. However, the formation of secondary caries around dental crowns can be observed even with no marginal discrepancies [111]. To overcome this clinical challenge, contact-killing materials may provide a solution to prevent the attachment of microorganisms and the subsequent onset of caries. Using a resin-based crown cement containing 5% DMAHDM was effective in reducing the *S. mutans* biofilms by approximately 2 logs. The formulation also contained NACP fillers as a remineralization strategy to complement the contact-killing surface of the cement. The same study revealed that incorporating DMAHDM and NACP did not adversely affect the bond strength or the mechanical properties of the cement [86]. More studies are needed in the future to elaborate on the behavior of such cement in luting dental crowns and fixed prostheses. 

## 6. Future Considerations of Contact-Killing Materials

Many studies in the dental literature have reported the use of the contact-killing approach for resin-based materials and have demonstrated promising results to manage the growth of biofilm over the dental materials.

Most of the in vitro studies reporting the antibacterial action of contact-killing materials did not comprehensively evaluate clinically relevant mechanical properties. For example, most of the studies on composite restorations reported mechanical tests such as flexural strength and elastic modulus. Other mechanical properties, such as water sorption, solubility, fatigue, and compressive strength, are rarely documented. One example can be observed with dental adhesives, where some studies reported the shear bond strength but not the micro-tensile bond strength. A significant amount of literature has demonstrated that micro-tensile bond strength correlates more closely with the clinical behavior of the material and is, therefore, more relevant. Performing the complete set of mechanical and physicochemical tests is essential to obtain reliable information about the performance of such materials and drive the clinical investigations of these materials.

Another point to consider is related to the surface properties of the dental materials over time. As restorative materials suffer alterations under mechanical challenges, such as toothbrushing, wear, and abrasion, surface roughness changes can occur along with the clinical service of polymeric restorations [112].

Crucial information about the degree of conversion, depth of cure, polymerization kinetics, and shrinkage were missing in the designed contact-killing materials. Resin-based materials with a low degree of conversion and depth of cure could be associated with a high percentage of uncured monomers that may leach, inducing a cytotoxic reaction to the surrounding tissues and promoting bacterial attachment on the material [62]. Materials with a low degree of conversion could also have inferior mechanical properties and reduced longevity [113]. Assessment of polymerization shrinkage is also crucial as it may increase the risk of bacterial invasion at the tooth–restoration interface. Obtaining more information in regard to the polymerization of such formulations is essential to predict the success of these materials.

Long-term evaluation of dental materials is necessary to ensure clinical longevity. This type of evaluation can encompass both mechanical and antibacterial properties. Contact-killing materials may have long-lasting therapeutic effects compared to materials with release-behavior, since the release of such ions may affect the stability and durability of the material and reduce the antibacterial effectiveness overtime. Moreover, the induction of bacterial resistance of contact-killing materials needs to be assessed [114,115]. There are no reported studies concerning the development of antibacterial resistance against QAMs. However, some studies found that QAMs did not induce bacterial resistance after the exposure to many passages of dental pathogens, which may indicate the benefits of QAMs to target dental biofilms [116,117].

Contact-killing materials are also susceptible to reduced effectiveness over time due to aging and exposure to mechanical and chemical degradation. These factors may reduce the mechanical properties and also induce leaching of the resin composite monomers, which may decrease the antibacterial action of the material. As a result, evaluating the long-term mechanical and antibacterial properties of contact-killing materials in restorative dentistry is needed to assure the longevity of such materials.

The use of translational (intraoral in situ & animal) models to test contact-killing materials inside the oral cavity is an important method to assess the clinical viability of new materials. The oral environment is quite different compared to laboratory settings. The diversity of the oral microbiome, salivary flow, and acidity affect the formation of biofilm and the performance of restorative materials inside the mouth. Therefore, dental researchers are highly encouraged to apply in situ models to test these materials inside the oral cavity as a translational assessment of these materials. When assessing the long-term effectiveness of various antimicrobial strategies via these translational models, contact-killing resin-based materials have been shown to induce sustained antibacterial action, clinically acceptable mechanical and physical properties, good polymerization behavior, and low cytotoxicity.

## 7. Conclusions

Restorative resin-based materials may prematurely fail due to bacterial colonization and the formation of pathogenic biofilm. Developing non-leaching contact-killing strategies could improve the clinical performance and longevity of such materials. Several studies have demonstrated the ability of non-leaching polymeric restorative materials to suppress oral disease-related pathogens in vitro. The addition of photopolymerizable quaternary ammonium-based monomers that can covalently bond to resin-based dental materials has shown a strong antibacterial effect that does not diminish over time.

Furthermore, the quaternary ammonium-based resins do not limit the antibacterial effect to the surface only. These materials are antibacterial throughout the entire volume. For a dental material with an antibacterial surface coating, occlusal wear may remove the surface layer, and the antibacterial effect may be lost. However, materials with quaternary functionality through the entire volume retain the same antibacterial function even after surface wear. Further studies are needed to examine the long-term antibacterial efficacy and their mechanical stability over time. The use of translational models to test these compounds in conditions simulating the oral cavity is also needed to support clinical performance and develop new applications for such materials.

## Figures and Tables

**Table 1:** The most commonly used quaternary ammonium monomers and their application in restorative dentistry.

Commonly used Quaternary Ammonium Monomers (QAMs)	Resin-Based Dental Material	Clinical Application
MDPB [63]	Resin composites	Restoration of defective tooth structure.
DMAHDM [60,64,65,66,67]		
IDMA1 [68]		
MDPB [69,70]	Dental adhesives	Used as an interface to bond the composite resin restoration to the tooth structure.
DDMAI [71]		
IDMA1 [71]		
IDMA2 [71]		
DMAE-CB [71]		
DMAHDM [72]		
QPEI [73,74]	Root canal sealers	Used with gutta-percha to obturate and seal the root canal system and prevent future leakage.
MDPB [75]		
DMAHDM [35,76,77]		
DMAHDM [78,79,80]	Dental Sealants	Used to occlude teeth anatomic features that facilitate plaque accumulation.
TAT [81]		
METAC [82]		
DMADDM [83]	Denture-base materials	Part of prosthetic appliances which rests on the oral mucosa and carries artificial teeth.
MAE-HB [84]	Orthodontic adhesives/cements	Used to bond orthodontic brackets to the tooth structure.
DMAHDM [85]		
DMAHDM [86]	Crown cements	Used to bond dental crowns/bridges to the tooth structure.

MDPB, 12-methacryloyloxydodecylpyridinium bromide; DMAHDM, Dimethylaminohexadecyl methacrylate; DDMAI, 2- dimethyl-2-dodecyl-1-methacryloxyethyl ammonium iodine; IDMA1, 2-methacryloyloxyethyl dimethylammonium; IDMA2, 2,2-bis(methacryloxyloxyethyl dimethylammonium); DMAE-CB, Methacryloxylethyl cetyl dimethyl ammonium chloride; QPEI, Quaternary ammonium polyethylenimine; TAT, 1,3,5-tri acryloyl hexahydro-1,3,5-triazine; METAC, (2-(methacryloyloxy)ethyl) trimethylammonium chloride; DMADDM, Dimethylaminododecyl methacrylate; MAE-HB, 2-methacryloxylethyl hexadecyl methyl ammonium bromide.
